# Mean Temperature Loss During General Anesthesia for Laparoscopic Cholecystectomy: Comparison of Males and Females

**DOI:** 10.7759/cureus.17128

**Published:** 2021-08-12

**Authors:** Usama Ahmed, Hameed Ullah, Khalid Samad

**Affiliations:** 1 Anesthesiology, Pain Medicine, The Aga Khan University, Karachi, PAK; 2 Anesthesiology and Critical Care, The Aga Khan University, Karachi, PAK; 3 Anesthesia and Critical Care, The Aga Khan University, Karachi, PAK

**Keywords:** general anesthesia, laparoscopy, cholecystectomy, subcutaneous fat, body temperature, hypothermia, male, female

## Abstract

Introduction

Mild hypothermia is common after general anesthesia. It is associated with discomfort and shivering. Greater fall of temperature is associated with more devastating complications. Data regarding the effect of gender on perioperative hypothermia is scanty.

Objectives of the study

To determine and compare mean core temperature loss in males and females undergoing laparoscopic cholecystectomy under general anesthesia.

Setting and design

Descriptive cross-sectional study in a tertiary care teaching hospital.

Subjects and methods

Ninety-seven elective laparoscopy patients were included through non-probability consecutive sampling. Intraoperatively, there was standardization of monitoring equipment, drapes, operation room temperature (21-22 °C), humidity (50%), irrigation fluid temperature (37 °C), peritoneal CO_2_ temperature (21-22 °C), anesthetic fresh gas flow rates at induction and maintenance. Temperature recording equipment (nasopharyngeal probe) and temperature recording interval (10 minutes) were also standardized from induction till the end of surgery. Final temperature was recorded at the end of surgery before emergence.

Results

Mean temperature loss was 0.73 ⁰C ± 0.47⁰C. Mean loss was significant in males compared to females with a mean difference of 0.28°C ± 0.93⁰C; P-value= 0.003.

Conclusion

Mean temperature decreases significantly in laparoscopic cholecystectomy patients under general anesthesia. We recommend that more care is needed to prevent hypothermia in male patients because of their higher susceptibility to hypothermia.

## Introduction

Temperature is lost invariably under general and regional anesthesia. It has adverse effects on the outcomes of patients. Hypothermia prevalence has been reported 27.1% and 23.8% in male and female patients, respectively [1].

Risk factors for hypothermia are preoperative hypothermia, age extremes, burns, trauma, fluid shifts, and prolonged duration of surgery. Protective structures against temperature loss are muscles, fat and skin [2]. Subcutaneous fat regulates temperature due to its property of physical insulation [3]. It is more in females as compared to males [4]. Data regarding the effect of gender on hypothermia is scanty.

The objective of the study was to determine mean temperature loss in males and females undergoing laparoscopic cholecystectomy in general anesthesia. Secondary objective was to compare mean temperature loss between males and females.

## Materials and methods

The research was carried at preoperative area, and operating rooms (OR) after obtaining approval from hospital Ethical Review Committee (4779-Ane-ERC-17). Duration of the study was six months from January, 2019 to June, 2019. Sampling technique was non-probability consecutive. Research design was cross-sectional descriptive type.

Temperature recordings were done by the primary anesthesiology team who was involved in the perioperative anesthetic management of the case and not involved in the study credits.

We included 97 (38 male and 59 female) patients in the study after obtaining informed consent. Inclusion criteria were: age between 18 and 50 years, American Society of Anesthesiologists (ASA) 1 & 2 patients and elective laparoscopic cholecystectomies. Exclusion criteria included emergency surgery, surgery time more than two hours, laparoscopic converted to open cholecystectomy, history of fever in the last one week, refusal to participate in study, patients on antibiotics &/or antipyretics, and intraoperative temperature variation of 2.5°C from baseline temperature.

A warmed (37⁰C) mattress was placed on the operating table before the arrival of the patients. Intraoperative monitoring was as per American Society of Anesthesiologists standard one. This included recording of electrocardiogram, peripheral oxygen saturation, blood pressure, and end-tidal capnograph throughout the anesthetic phase. An intravenous line was established using warmed Lactated Ringer's or 0.9% NaCl solution depending on patient's characteristics. These fluids were stored at room temperature. Temperature of BW-685 infusion warmer by Biegler® GmbH Austria was set at 37°C. Choice of anesthetic drugs for general anesthesia was left to the primary anesthesiologist. HME filter by Intersurgical® Limited, Wokingham, Berkshire, UK was used distal to the ‘Y’ limb of anesthesia circuit. Following the induction of anesthesia, a nasopharyngeal temperature probe (Adult 400-Series by Vayire™) was placed. Its position behind the soft palate was confirmed by the surface marking between 9 to 10 cm. Final probe tip position was confirmed by C-MAC ® video laryngoscope (Karl Storz, Tuttlingen, Germany) for proper site placement. Environment of the operating room was standardized as well. OR temperature was maintained between 21 - 22⁰C and humidity was set at 50%. Temperature of patients was monitored from induction till emergence at an interval of ten minutes along with all other monitoring. Fresh flow of gas was set at two liters/minute during anesthesia maintenance and 10 liters/minute at induction and emergence. Maintenance flow rates are standardized at our department due to teaching purposes.

All patients had the same disposable surgical drapes. For pneumoperitoneum, CO_2_ was used from the hospital wall hose and its temperature is kept constant at 21-22⁰C by the engineering department of the hospital. Variable flow of gas was used to maintain an intra-abdominal pressure of less than 15 mmHg.

**Figure 1:**
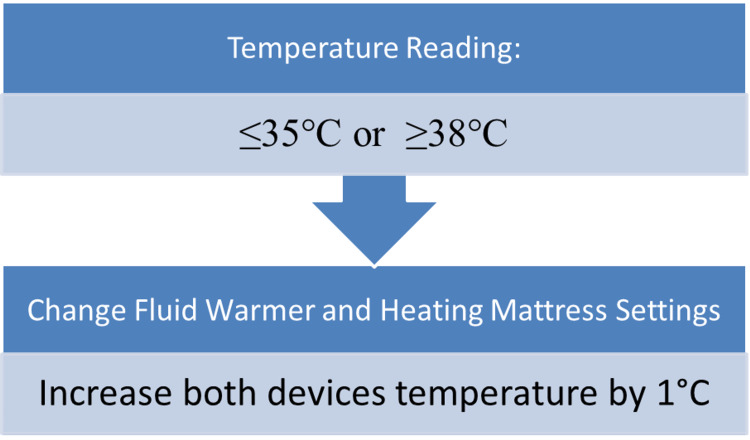
Algorithm for temperature adjustment.

Intraoperatively, temperature of the OR table mattress and intravenous fluid warmer was adjusted as follows: If the patient’s temperature dropped to 35⁰C, temperature of both devices had to be increased by 1⁰C and if the patient’s temperature rose above 38⁰C, it had to be decreased 1⁰C to prevent adverse effects (Figure 1).

At the end of the procedure, patients anesthetics were discontinued, neuromuscular blockade was reversed, final reading of temperature was obtained and trachea extubated. They were then transferred to post-anesthesia care unit (PACU).

Sample size calculation

Sample size calculation was based on previous study by Bessell et al, who reported that mean difference in temperature drop between males and females was 0.5°C (standard deviation = 0.8°C) [5]. A prospective Brazilian longitudinal cohort study found lifetime prevalence of cholecystectomy to be 4%. 5.3% of females underwent cholecystectomy whereas in males it was found to be 2.4% [6]. Since females undergo laparoscopic cholecystectomy 1.5 to 2 times more, sample allocation ratio was set as 1.5. 97 patients (38 male and 59 females) were recruited to detect a change in temperature of 0.5°C with 80% power and 5% type I error.

Statistical analysis

A statistical analysis was performed using Statistical Package for Social Science (SPSS ver-19, Inc., Chicago, IL, USA). The normality of outcome distribution was determined by the Kolmogorov-Smirnov or Shapiro-Wilk test. Categorical point estimation was reported in terms of frequency and percentage for ASA-status, cholecystectomy. Numeric point estimation was reported in terms of mean (standard deviation) or median (25th-75th percentile) for age, weight, height, BMI, temperature. Since the temperature was the primary endpoint of this study, mean values with standard deviation were calculated. Unpaired t-test was used to compare mean temperature between males and females. General linear model was used to control the effect of BMI and to observe the difference between male and female temperature. Regression coefficient and 95% confidence interval were reported. A P-value ≤ 0.05 (two-sided) was considered significant.

## Results

Ninety-seven patients were recruited for the study from January 2019 to June 2019. None of the patients were excluded from study. Demographic details of the study population are shown in Table 1 and temperature recordings are shown in Table 2. No adverse perioperative surgical or anesthetic events occurred. 

**Table 1:** Demographics of study participants (n = 97). SD: standard deviation; BMI: body mass index.

Characteristic	Males (n = 38)		Females (n = 59)	
	Mean	SD	Mean	SD
Age (years)	39.55	8.78	39.20	8.10
Weight (kg)	70.90	11.51	63.61	9.92
Height (meters)	1.67	0.13	1.58	0.08
BMI (kg/m^2^)	24.96	3.52	25.38	3.51
Duration of surgery (minutes)	91	26	86	21

**Table 2:** Mean temperature at intervals.

Time from induction (minutes)	Male		Female		P-value
	n	Mean temperature (°C)	n	Mean temperature (°C)	
0	38	36.38 ± 0.26	59	36.436 ± 0.29	0.339
10	38	36.21 ± 0.26	59	36.246 ± 0.29	0.567
20	38	36.07 ± 0.28	59	36.117 ± 0.32	0.476
30	38	35.97 ± 0.27	59	36.034 ± 0.33	0.315
40	38	35.88 ± 0.29	58	35.964 ± 0.37	0.215
50	37	35.78 ± 0.28	55	35.880 ± 0.40	0.175
60	37	35.71 ± 0.28	49	35.811 ± 0.39	0.199
70	34	35.56 ± 0.32	31	35.810 ± 0.39	0.007
80	27	35.54 ± 0.32	28	35.775 ± 0.40	0.022
90	23	35.40 ± 0.30	25	35.724 ± 0.39	0.002
100	14	35.43 ± 0.28	18	35.628 ± 0.28	0.050
110	8	35.40 ± 0.3	13	35.715 ± 0.26	0.028

Mean temperature loss was 0.73°C ± 0.47⁰C. Temperature loss occurred gradually, and lowest temperature was recorded at 100 minutes from induction which was 0.84⁰C. Afterwards, temperature increased 0.2⁰C till 110 minutes. In males, mean loss was 0.9°C and in females 0.61°C. Loss was significantly high in males as compared to females (mean difference = -0.28⁰C ± 0.93⁰C; P-value = 0.003).

Plausible factors having effect on temperature were analyzed. Considering age, patients older than 40 years were compared to those who were less than equal to 40 years. Although older age group lost more temperature but it was not statistically significant (p=0.188) .However, males lost 0.45⁰C more as compared to females (P = 0.002, Table 3).

In terms of weight, highest temperature loss was 0.68⁰C in 61-70 Kg patients. In this category, males lost 0.41⁰C more than females (P-value: 0.047). Similarly, male patients with BMI 25-29 kg/m^2^ lost 0.32⁰C more than females (P = 0.04).

More than 75 liters CO_2_ used for the Pneumoperitoneum produced more temperature loss as compared to ≤75 Liters (Table 3). Highest temperature loss was in the patients receiving 77-155 liters. Males lost 0.56⁰C more than females (P = 0.008). Height was not found to have any effect on temperature loss.

**Table 3:** Stratification analysis showing comparison of mean core temperature loss (n = 97). Note. *P < 0.05, ***P: 0.001.

Variables	Overall				P-value	Males		Females	P-value (males vs females)
Age group									
≤ 40 Years		-0.67±0.41		0.188		-0.73±0.43		-0.63±0.41	0.394
>40 Years		-0.79±0.51				-1.06±0.41		-0.61±0.49	0.002***
BMI (kg/m^2^)									
≤25	-0.80±0.35			0.122		-0.90±0.42	-0.73±0.28		0.110
>25	-0.66±0.55					-0.91±0.48	-0.51±0.54		0.013*
Fresh flow of gases (liters/minute)									
≤75		-0.71±0.44	0.655			-0.81±0.42		-0.62±0.45	0.145
>75		-0.75±0.49				-1.02±0.46		-0.61±0.46	0.005***

Effect of gender and BMI on temperature loss is shown in Table 4.

**Table 4:** Effect of sex and BMI on temperature loss using general linear model. *P < 0.05.

Parameter	Regression Coefficient	Std. Error	P-Value	95% confidence interval	
				Lower bound	Upper bound
Gender					
Male vs. female	-0.244	0.093	0.010*	-0.428	-0.06
BMI					
≤25	-0.363	0.139	0.010*	-0.638	-0.088
25.1 to 29.9	-0.31	0.144	0.033*	-0.596	-0.025
≥30	0				
Intercept	-0.339	0.122	0.006	-0.581	-0.097

## Discussion

Human temperature is kept in a narrow range of 36.5°C-37.3°C by physiological homeostasis. At the cellular level, hypothermia freezes cytoplasm and results in crystals formation which results in cellular rupture. There is a decrease in microcirculation due to increase in blood viscosity along with increased coagulation [7]. Lethal triad is a serious consequential complication which includes hypothermia, acidosis and coagulopathy. Hypothermia is directly related to injury severity score and is independently related to multi-organ failure from metabolism [8]. According to a national study done in China, inadvertent intraoperative hypothermia incidence was between 4% and 90% [9].

We found mean temperature loss of 0.73⁰C in patients undergoing laparoscopic cholecystectomy. The results are similar to study by Makinen et al., who reported temperature decrease of 0.7⁰C in laparoscopic cholecystectomy [10]. Natalí et al. conducted a study on hypothermia in laparoscopic cholecystectomies. They found greater mean loss of 0.8°C and 1.3°C with water circulating warmer and forced air warmer, respectively [11].

Two main mechanisms of heat loss due to anesthesia are: disorganized thermoregulation due to anesthetic drugs and radiation of heat to operation room at lower temperature (18-24⁰C) [12]. Due to influence of anesthetic agents, temperature regulation interthreshold range is increased 10 times from its baseline (0.3°C-4°C) and other physiologic heat loss protection mechanisms are lost as well. Regional and local anesthetic techniques also cause heat loss [13]. Painful stimulation increases vasoconstriction threshold on its own [14]. All of these physiologic derangements lead to intra-operative hypothermia. There are many diseases as well that predispose to hypothermia like hypothyroidism, rheumatoid arthritis, hypopituitarism, liver disease and malnutrition. These disease states were not accounted for formulation of this study.

Surprisingly, temperature loss in laparoscopic procedures is more than in major open surgical techniques. Castillo et al. found that patients undergoing laparotomy for cholecystectomy lost 0.2°C at 80th minute from induction, while those undergoing laparoscopic removal lost 0.43°C for the same time [15]. This temperature loss is profoundly significant for a minimally invasive surgical procedure of laparoscopic cholecystectomy which has a usual duration of only 0.5-2 hours only. Temperature loss is due to several reasons like anesthetics, insufflation of CO2, use of intra-abdominal irrigation fluid and the operating room environment.

A mean difference of 0.28°C in temperature loss between males and females was noted in the study. Considering age, BMI and the amount of CO2 used for pneumoperitoneum, males lost more temperature than females. The loss was 0.45°C more compared to female patients group older than 40 years. Using the general linear model, P-value of 0.01 was obtained when males were compared to females reflecting greater susceptibility for hypothermia (Table 4). Sagiroglu et al. identified male sex as a risk factor for perioperative temperature loss during major abdominal surgeries [16]. Similar results were found by Panagiotis et al. who reported the length of stay in PACU of male patients longer than females due to postoperative hypothermia [17]. A study by Kim conducted on abdominal surgeries refuted our findings. They depicted no correlation between an individual's gender and general anesthesia [18].

Despite the many differences in organ systems between the genders, there is difference in amount of subcutaneous fat among males and females which deserves attention. It serves as a thermal source and shock absorber. In adults, one-third of the total body weight consists of subcutaneous fat. Kongsayreepong et al. investigated the predictive factors against post-operative hypothermia of post general surgical patients at the time of intensive care unit admission and found that increased body weight was one of the protective factors [19].

In our study, when BMI was analyzed to have an effect on temperature loss, patients with BMI ≤29.9 kg/m^2^ had significant loss of temperature (Table 4). Yi et al. identified BMI ≥ 25 kg/m^2^ as a protective factor against temperature loss in a national study on Chinese population regarding intraoperative hypothermia [9]. This explains some role of fat in the human temperature dynamics. Subcutaneous fat differences are said to be due to impact of sex hormones and aging process. Estrogen influences adipocytes due to its influence on the enzyme: lipoprotein lipase. Higher subcutaneous fat in females compared to males is attributed to higher caloric requirements of females as in pregnancy and lactation. Female hypodermis is eight percent thicker than males [20].

Role of subcutaneous fat in temperature maintenance is not new to anesthesiology. It has been well established in pediatric anesthesia. Heat loss by conduction and radiation is attributed to greater loss in children due to lesser subcutaneous fat. Although there are other factors as well in children like higher surface area to volume ratio, immature epidermal barrier, larger surface area and narrow range of heat production from metabolism [21]. The effect is so pronounced that even in non-surgical office-based procedures like MRI, there’s significant temperature loss due to general anesthesia. This was observed in a study by Ruth et al. [22].

We found that age group older than 40 years lost more temperature compared to less than 40 years old group. Correlation between advanced age and perioperative hypothermia has been supported by Ott and Kim in their respective studies [23,24]. Chun et al. also reported similar results [25]. Various reasons for the greater temperature loss as reported in the literature are: increased ratio of body weight to body surface area, decreased amount of subcutaneous fat and decreased metabolism [16]. Wetz et al. analyzed seven prospective studies investigating core temperature and found that 21.3% patients were hypothermic before induction of anesthesia. Male and older patients were found to be at greater risk [26].

CO2 used for pneumoperitoneum especially at high-flow rates over a prolonged period results in a significant fall in core temperature as reported by Bessell et al. and Figueredo-Gaspari [5,27]. Hypothermic stress of -0.3°C is produced by carbon dioxide insufflation for creating pneumoperitoneum. Therefore, warming of the carbon dioxide for abdominal insufflation was recommended by Ott to counteract hypothermia [23]. It was not a control factor in our study since the amount of CO2 used varied from patient to patient. Irrigation fluid was not correlated in our study because of volume variation between patients and our limited study goals.

Perioperative temperature change has a chain of events which consumes time. Temperature loss in anesthesia occurs classically in redistribution, linear and plateau phases. Temperature increase was also noted in some of our patients after achieving plateau. We recommend longer duration surgeries to be investigated to yield more scientific information regarding temperature dynamics over time. Anesthetic depth and pharmacologic variables were not correlated in the study which should be accounted for future research. Another limitation of the study was the temperature recording equipment. We used nasopharyngeal probe due to non-cardiac surgery. Core temperature should be checked for further precision by means of PA Catheter. Female reproductive cycle was not accounted for the temperature change as it causes elevation of 0.3⁰F (0.1⁰C) at the time of ovulation, but this temperature change is not large enough to deviate the results of the study significantly [28]. Surgical draping was standardized and carefully placed not to leave any area exposed. Theoretically, there would be temperature loss due to radiation of heat from unwanted non-insulated areas. We assume that its effect on major heat loss would be less compared to other major factors. Therefore, we recommend trial of absolute physical conditions for animal study models.

## Conclusions

There is significant temperature loss in laparoscopic cholecystectomy. We postulate that there is more male vulnerability for hypothermia compared to females under general anesthesia which may be attributed to lesser subcutaneous fat. Since normothermia maintenance is a basic consideration in anesthetic management of a patient, this study can be extrapolated to consider the differences in organ systems between males and females for better anesthetic management for the surgical and non-surgical cases.
